# Allocation of Nutrients and Leaf Turnover Rate in Poplar under Ambient and Enriched Ozone Exposure and Soil Nutrient Manipulation

**DOI:** 10.3390/biology13040232

**Published:** 2024-03-31

**Authors:** Elena Paoletti, Mario Pagano, Lu Zhang, Ovidiu Badea, Yasutomo Hoshika

**Affiliations:** 1 IRET-CNR, Via Madonna del Piano 10, Sesto Fiorentino, 50019 Florence, Italy; elena.paoletti@cnr.it (E.P.); yasutomo.hoshika@cnr.it (Y.H.); 2 College of Landscape and Architecture, Zhejiang A&F University, 666 Wusu Street, Hangzhou 311300, China; caszhanglu@hotmail.com; 3 National Institute for Research and Development in Forestry ‘Marin Drăcea’, 128, Eroilor Bvd., 077190 Voluntari, Romania; ovidiu.badea63@gmail.com; 4 Faculty of Silviculture and Forest Engineering, Transilvania University, 1, Ludwig van Beethoven Street, 500123 Brasov, Romania

**Keywords:** ozone, poplar, soil nutrient, leaf turnover

## Abstract

**Simple Summary:**

The study observed how plants adjust leaf turnover rates and nutrient allocation at the organ level to counter O_3_ damage. Various O_3_ treatments (ambient concentration, 1.5 × AA, 2.0 × AA) and fertilization levels (N: 0 and 80 kg N ha^−1^ y^−1^; P: 0 and 80 kg N ha^−1^ y^−1^) were applied to an O_3_-sensitive poplar clone in a FACE experiment. The results revealed significant effects of both fertilization and O_3_ on nutrient content, with increases in foliar C and N (+5.8% and +34.2%) and root Ca and Mg (+46.3% and +70.2%). Accelerated leaf turnover rates due to O_3_ exposure were observed, indicating its significant role in this physiological parameter. O_3_ fumigation influenced the overall allocation of primary and secondary elements across plant organs. These findings underscore the ecological implications of altered element allocation in plant leaves in response to elevated O_3_ levels.

**Abstract:**

An excess of ozone (O_3_) is currently stressing plant ecosystems and may negatively affect the nutrient use of plants. Plants may modify leaf turnover rates and nutrient allocation at the organ level to counteract O_3_ damage. We investigated leaf turnover rate and allocation of primary (C, N, P, K) and secondary macronutrients (Ca, S, Mg) under various O_3_ treatments (ambient concentration, AA, with a daily hourly average of 35 ppb; 1.5 × AA; 2.0 × AA) and fertilization levels (N: 0 and 80 kg N ha^−1^ y^−1^; P: 0 and 80 kg N ha^−1^ y^−1^) in an O_3_-sensitive poplar clone (Oxford: *Populus maximowiczii* Henry × *P. berolinensis* Dippel) in a Free-Air Controlled Exposure (FACE) experiment. The results indicated that both fertilization and O_3_ had a significant impact on the nutrient content. Specifically, fertilization and O_3_ increased foliar C and N contents (+5.8% and +34.2%, respectively) and root Ca and Mg contents (+46.3% and +70.2%, respectively). Plants are known to increase the content of certain elements to mitigate the damage caused by high levels of O_3_. The leaf turnover rate was accelerated as a result of increased O_3_ exposure, indicating that O_3_ plays a main role in influencing this physiological parameter. A PCA result showed that O_3_ fumigation affected the overall allocation of primary and secondary elements depending on the organ (leaves, stems, roots). As a conclusion, such different patterns of element allocation in plant leaves in response to elevated O_3_ levels can have significant ecological implications.

## 1. Introduction

Element stoichiometry considers the balance between nutrients and how environmental factors affect it [1]. “Stoichiometric homeostasis” is the degree to which organisms maintain a constant elemental composition in response to the availability of environmental resources [2]. Plant stoichiometry has traditionally focused on the three primary macronutrients, i.e., carbon (C), nitrogen (N), and phosphorus (P) that are quantitatively more important, while the other macronutrients, i.e., potassium (K), calcium (Ca), sulfur (S), magnesium (Mg), have been less studied although they are essential for plant growth. Ågren and Weih [1] found that N and P are the driving elements in plant stoichiometric relations and other elements scale with respect to them. For understanding how plants respond to environmental stressors, the allocation of nutrient elements to different plant organs has to be considered [3] as they may show distinct patterns of C:N:P stoichiometry [4]. Interestingly, stoichiometric homeostasis for N and P was found among vegetative tissues of forest plants in natural communities [5]. Excess N [6], O_3_ [7], and P [8] are now stressing our plant ecosystems, but their combined impacts on multi-elemental stoichiometry at the organ level are unknown. Nitrogen deposition due to anthropogenic N emissions is still increasing [9], with peaks of 80 kg N ha^−1^ y^−1^ in some areas in the northern hemisphere [10,11]. Since nitrogen oxides are among the main ozone (O_3_) precursors, O_3_ exposure and N deposition usually co-exist [12]. Soil acidification induced by excess N deposition may cause P limitation for tree growth [13]. Atmospheric P deposition over time is relatively constant [14], while inputs are mostly from wastewater and agriculture [8] with soil P availability ranging from 0.5 to 1.3 g kg^−1^ depending on soil type [15]. The individual impacts of O_3_, N, and P on plant stoichiometry have been well investigated. Ozone exposure usually increased the foliar concentration of macronutrients, while C did not vary (e.g., [16,17,18]), although inconsistent responses (e.g., [19]) or decreases [20] were also observed. When different plant organs were investigated, O_3_ exposure induced decreases [21] or minimal changes in C allocation to roots [22]. In the only study that investigated O_3_ exposure with N fertilization, O_3_ increased foliar Ca concentration without interactive effects [23]. The stoichiometric responses to concurrent N and P additions have been largely investigated and showed that P addition without N addition increases foliar N concentrations, whereas P addition with N addition does not have an effect [24]. Tian et al. [25] reviewed the stoichiometric relations in plants subject to nutrient additions, elevated CO_2_ and temperature, or decreasing precipitation, and found that N additions into soils increased plant N relative to other elements, experimental warming tended to decrease plant N and P, decreased precipitation increased plant N:P ratio in experiments and decreased it along natural gradients, and responses to elevated CO_2_ varied with the element so that plant N:Mg and P:Mg increased, while N:Mn and P:Mn decreased. They concluded that environmental changes altered plant stoichiometric couplings between N and P vs seven other mineral elements (K, Ca, Mg, S, Mn, Fe, and Al), but our understanding of the impact of co-occurring stressors on plant element stoichiometry is still largely imperfect. The turnover rate of leaves affects the proportion of younger leaves on a plant, which are known to have higher rates of photosynthesis [26]. Leaf turnover rate can also be linked to an increase in nutrients. For example, according to Aerts [27], increased stem mortality resulted in an increased leaf turnover per unit ground area with increasing nutrient availability in the soil due to more material being released. However, it is still unclear how O_3_ can alter leaf turnover rates in numerous species under different soil N and P conditions. We aimed to experimentally untangle the complex impacts of the co-occurring N, O_3_, and P factors on poplar stoichiometric relations in different plant organs. Poplar was selected because it is widely used for wood production and is considered as a model system in plant biology [28]. The study utilized an O_3_-sensitive clone of poplar [29,30]. Our main objective was to investigate the effects of O_3_ and fertilization for the first time on primary and secondary nutrient allocation and leaf turnover in poplar during a New-Generation 3D Ozone FACE (Free Air Controlled Exposure) experiment. In particular, we tested the hypothesis that elevated levels of O_3_ primarily modify nutrient uptake from the soil to adjust nutrient concentrations and maintain stoichiometric homeostasis.

## 2. Materials and Methods

### 2.1. Plant Material and Treatments

One-year-old rooted cuttings of the O_3_-sensitive Oxford clone (*Populus maximoviczii* Henry × *P. berolinensis* Dippel) were raised in 10-l pots with sand:peat:soil = 1:1:1 (*v*:*v*:*v*). From 1 May to 1 October, the cuttings were exposed to three levels of O_3_ exposure in a free-air controlled exposure (FACE) facility, i.e., ambient air (AA), 1.5 AA and 2.0 AA. A mixture of ambient air and O_3_ generated by a TGOC13X generator (Triogen Ltd., Glasgow, UK) was delivered via a network of Teflon tubes hanging down from a fixed grid above the cuttings. Ozone concentration at plant height was monitored by Model 202 analyzers (2B Technologies Inc., Boulder, CO, USA). Further details on the FACE facility are in Paoletti et al. [31]. Three replicated 25 m^2^ blocks were assigned to each O_3_ concentration, with 18 cuttings in each block. Three plants in each block were randomly assigned to one of the following nutritional treatments: N0-P0 (0 kg N ha^−1^; 0 kg P ha^−1^), N0-P80 (0 kg N ha^−1^; 80 kg P ha^−1^), N80-P0 (80 kg N ha^−1^; 0 kg P ha^−1^), and N80-P80 (80 kg N ha^−1^; 80 kg P ha^−1^). Nitrogen was supplied as NH_4_NO_3_ (0 and 5.0 mM solutions) according to Thomas et al. [32], and simulated realistic deposition [10,11]. Phosphorus was supplied as KH_2_PO_4_ (0 and 1.0 mM solutions) according to Lewis and Strain [33] and simulated a realistic range of soil available P [15]. Nitrogen and P concentrations in soils were shown in a previous paper from the same experiment [13] and were 1.7 ± 0.1 g N kg^−1^ in N0, 2.7 ± 0.1 g N kg^−1^, 0.5 ± 0.1 g P kg^−1^ in P0 and 1.0 ± 0.1 g P kg^−1^ in P80. In detail, 200 mL of NH_4_NO_3_ or KH_2_PO_4_ solutions with the different concentrations described above were added into the potted soil every 2–3 days along the entire growing season in order to reach the following total amounts at the end of the experiment: N80: 392.5 mg N per cutting, P80: 392.5 mg P per cutting. Concurrently, KCl was supplied into the soil that did not receive KH_2_PO_4_ to keep an equal amount of K among treatments [34]. The pot position was changed every two weeks within each block to eliminate possible positional effects due to irrigation or light. Plants were irrigated to field capacity every 2–3 days. A detailed description of the experimental design is shown in [13], where we showed that O_3_ risk assessment is affected by the availability of N and P in the soil.

### 2.2. Sampling and Measurements

For all plants, we counted the number of attached leaves on 18 July, 29 August and 23 September. In addition, the number of fallen leaves was estimated by the number of leaf traces. According to [35], the leaf turnover rate per each plant was determined as follows:

Leaf turnover rate = 1/2 (NFL + NNL)/MNL/Days

where NFL and NNL are the numbers of fallen leaves and newly developed leaves, respectively, MNL is the number of leaves between two consecutive surveys (18 July to 29 August or 29 August to 23 September), and Days is the number of days between an assessment and the following one. In October, before the occurrence of any visible symptom of leaf senescence, all cuttings were harvested, divided into clean roots, stems (including shoots), and leaves, and dried at 103 °C in an oven until constant weight. Three randomized replicated 4 g dry weight (DW) sub-samples were collected for each combination of fertilization and O_3_ treatment, resulting in 36 samples for each plant organ. The samples were grounded in a mortar and subject to the following analyses. Total N content was determined using a modified Kjeldahl method [36]. Total P, K, Ca, S, and Mg contents were determined by inductively coupled plasma–optical emission spectroscopy (ICP-OES PerkinElmer Optima 2100 DV, Arcade, New York, NY, USA).

### 2.3. Statistical Analyses

The study was conducted using a replicated split-plot design, with two randomized factors: O_3_ treatment (AA, 1.5 × AA, 2.0 × AA) and fertilization treatment (N0-P0, N0-P80, N80-P0, N80-P80). Data were assessed for normal distribution using the Shapiro–Wilk test. A two-way ANOVA was conducted to evaluate the effect of O_3_ treatment with different fertilization treatments. Post-hoc differences were analyzed using the Tukey test (*p* < 0.05). GraphPad Prism v. 9 (GraphPad Software, Inc., Boston, USA) was used for all statistical analyses. To evaluate the interaction of all primary and secondary elements among the three plant organs, a Principal Component Analysis (PCA) was performed. The statistical software used for the PCA analysis was PAST v. 4.11 (PAleontological STatistics, Natural History Museum, University of Oslo).

## 3. Results

### 3.1. Primary Macronutrients (C, N, P, K)

**Figure 1:**
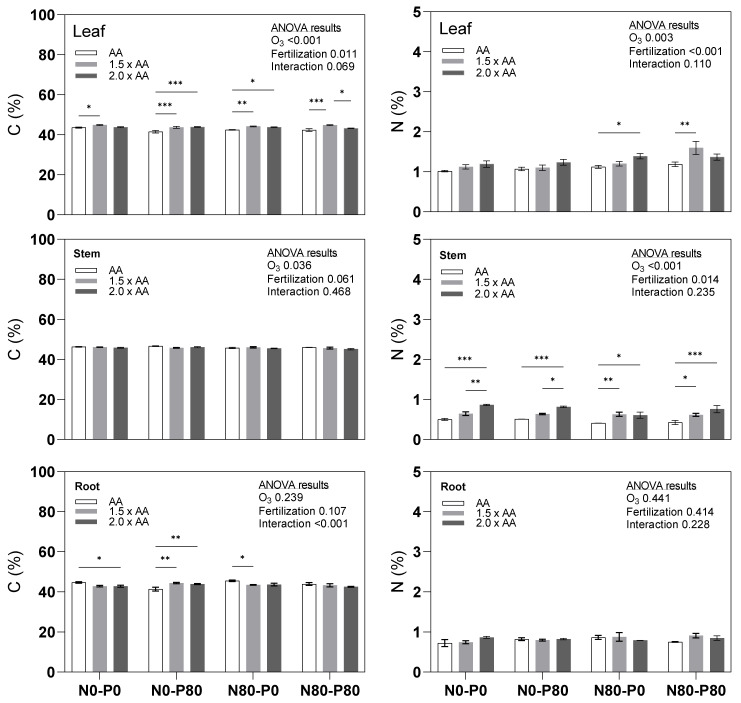
Primary macronutrients (C, N) in the leaves (Leaf), shoots-stems (Stem), and roots (Root) of Oxford poplar clone exposed to three levels of O_3_ (ambient air [AA], 1.5 × AA, 2.0 × AA) with different combinations of soil fertilization (two levels of N [0 and 80 kg N ha^−1^; N0 and N80] and two levels of P [0 and 80 kg P ha^−1^; P0 and P80]). The bars represent mean ± S.E. (*n* = 3). Asterisks indicate the level of significance of a 2-way ANOVA within each group: *** *p* < 0.001, ** *p* < 0.01, * *p* < 0.05. The absence of asterisks indicates that there were no significant differences (*p* > 0.05). The ANOVA table results explain the overall variation, with the level of significance expressed numerically through the *p*-value.

Elevated O_3_ levels had a noticeable impact on carbon (C) content, particularly in the leaves and stems, where it significantly increased compared to ambient air (AA). However, this effect was not observed in the roots (Figure 1). Notably, certain treatment combinations, such as N0-P0 (1.5 × AA), N0-P80 (1.5 × AA), N0-P80 (2.0 × AA), and N80-P80 (1.5 × AA), exhibited significantly higher foliar C content than AA (+3.0%, +5.3%, +5.8% and +5.7, respectively). Conversely, N0-P0 (2.0 × AA) and N80-P0 (1.5 × AA) displayed lower root C content than AA (−4.3% and −4.5%, respectively). The interaction between O_3_ and Fertilization for C content was significant only in the roots. The impact of elevated O_3_ levels on N content varied across different plant parts (Figure 1). While the average N content increased significantly in the leaves and stems under elevated O_3_ conditions compared to AA, there was no significant difference observed in the roots. Specifically, treatments like N80-P0 (2.0 × AA) and N80-P80 (1.5 × AA) demonstrated markedly higher foliar N content than AA (+23.7% and +34.2%, respectively). In addition, various treatment combinations showed significantly higher N content in the stems than AA. For instance, treatments such as N0-P0 (2.0 × AA), N0-P80 (2.0 × AA), N80-P0 (1.5 × AA), N80-P0 (2.0 × AA), N80-P80 (1.5 × AA), and N80-P80 (2.0 × AA) displayed increases by +72.1%, +60.5%, +54.6%, +48.5%, +45.6%, and +78.3%, respectively. Interestingly, no interaction was found between O_3_ exposure and fertilization treatments regarding N content.

**Figure 2:**
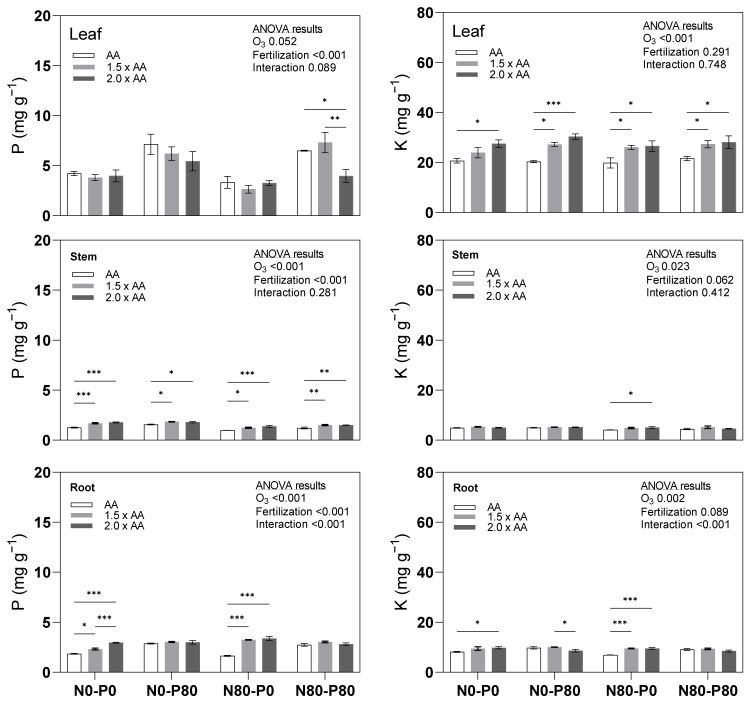
Primary macronutrients (P, K) in the leaves (Leaf), shoots–stems (Stem), and roots (Root) of the Oxford poplar clone exposed to three levels of O_3_ (ambient air [AA], 1.5 × AA, 2.0 × AA) with different combinations of soil fertilization (two levels of N [0 and 80 kg N ha^−1^; N0 and N80] and two levels of P [0 and 80 kg P ha^−1^; P0 and P80]). The bars represent mean ± S.E. (*n* = 3). Asterisks indicate the level of significance of a two-way ANOVA within each group: *** *p* < 0.001, ** *p* < 0.01, * *p* < 0.05. The absence of asterisks indicates that there were no significant differences (*p* > 0.05). The ANOVA table results provide an explanation of the overall variation, with the level of significance expressed numerically through the *p*-value.

Phosphorus (P) content exhibited significant variability among the treatments across leaf, stem, and root tissues (Figure 2). Notably, treatment N80-P80 (2.0 × AA) showed a substantial decrease in leaf P content by −38.9%. However, in the stem, results displayed significantly higher phosphorus content than AA. Treatments including N0-P0 (1.5 × AA), N0-P0 (2.0 × AA), N0-P80 (1.5 × AA), N0-P80 (2.0 × AA), N80-P0 (1.5 × AA), N80-P0 (2.0 × AA), N80-P80 (1.5 × AA), and N80-P80 (2.0 × AA) exhibited notable increases of +33.7%, +41.1%, +17.3%, +14.5%, +25.0%, +38.8%, +23.4%, and +23.4%, respectively. Similarly, in the root, treatments N0-P0 (1.5 × AA), N0-P0 (2.0 × AA), N80-P0 (1.5 × AA), and N80-P0 (2.0 × AA) showed significantly higher P content than AA, with substantial increases +25.2%, +59.9%, +97.2%, and +105.7%, respectively. Furthermore, the interaction between O_3_ exposure and fertilization treatments for P content was significant only in the root. Elevated O_3_ exposure had a significant impact on potassium (K) content across all plant organs, although the magnitude of the effect was comparatively lower in the stem compared to the leaf and root (Figure 2). In particular, results demonstrated significantly higher foliar K content than AA. Treatments such as N0-P0 (2.0 × AA), N0-P80 (1.5 × AA), N0-P80 (2.0 × AA), N80-P0 (1.5 × AA), N80-P0 (2.0 × AA), N80-P80 (1.5 × AA), and N80-P80 (2.0 × AA) with increases of +33.3%, +34.0%, +49.5%, +31.4%, +34.0%, +26.6%, and +30.3%, respectively. Furthermore, at the root level, treatments N0-P0 (2.0 × AA), N80-P0 (1.5 × AA), and N80-P0 (2.0 × AA) displayed significantly higher K content than AA (+19.7%, +37.5%, and +38%, respectively). Additionally, the interaction between O_3_ exposure and fertilization treatments for K content was significant only in the root.

### 3.2. Secondary Macronutrients (Ca, S, Mg)

**Figure 3:**
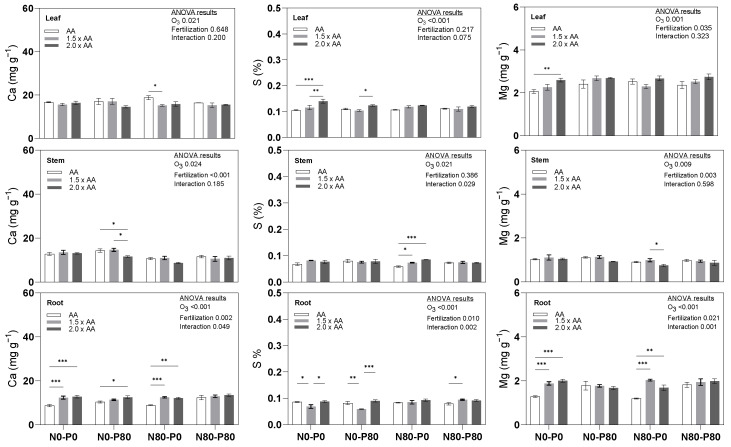
Secondary macronutrients (Ca, S, Mg) in the leaves (Leaf), shoots–stems (Stem), and roots (Root) of the Oxford poplar clone exposed to three levels of O_3_ (ambient air [AA], 1.5 × AA, 2.0 × AA) with different combinations of soil nutritional conditions (two levels of N [0 and 80 kg N ha^−1^; N0 and N80] and two levels of P [0 and 80 kg P ha^−1^; P0 and P80]). The bars represent mean ± S.E. (*n* = 3). Asterisks indicate the level of significance of a two-way ANOVA within each group: *** *p* < 0.001, ** *p* < 0.01, * *p* < 0.05. The absence of asterisks indicates that there were no significant differences (*p* > 0.05). The ANOVA table results explain the overall variation, with the level of significance expressed numerically through the *p*-value.

Treatment N80-P0 (1.5 × AA) exhibited significantly lower calcium (Ca) content in the leaf than AA (Figure 3), with a decrease of −19.0%. Similarly, in the stem, treatment N0-P80 (2.0 × AA) showed significantly lower Ca content than AA, with a reduction of −18.9%. Conversely, several treatment combinations displayed significantly higher root Ca content than AA. Treatments such as N0-P0 (1.5 × AA), N0-P0 (2.0 × AA), N0-P80 (2.0 × AA), N80-P0 (1.5 × AA), and N80-P0 (2.0 × AA) exhibited increases of +41.4%, +46.3%, +20.7%, +40.7%, and +36.6%, respectively. Furthermore, a significant interaction between O_3_ exposure and fertilization treatments was observed only in the root. Treatment N0-P0 (2.0 × AA) demonstrated a significantly higher foliar S content than AA (Figure 3), showing an increase of +33.6%. In the stem, treatments N80-P0 (1.5 × AA) and N80-P0 (2.0 × AA) displayed significantly higher S content than AA (+25.6% and +44.9%, respectively). Conversely, treatments N0-P0 (1.5 × AA) and N0-P80 (1.5 × AA) exhibited significantly lower root S content than AA, with decreases of −20.2% and −28.0%, respectively. However, treatment N80-P80 (1.5 × AA) showed a significantly higher root S content than AA (+19.3%). Additionally, significant interactions between O_3_ exposure and fertilization treatments for S content were observed in both the stem and root. Treatment N0-P0 (2.0 × AA) exhibited a significantly higher foliar magnesium (Mg) content than AA (Figure 3), showing an increase of +26.1%. Furthermore, in the root, treatments N0-P0 (1.5 × AA), N0-P0 (2.0 × AA), N80-P0 (1.5 × AA), and N80-P0 (2.0 × AA) displayed significantly higher Mg content than AA (+46.9%, +56.8%, +70.2%, and +41.1%, respectively). Additionally, a significant interaction between O_3_ exposure and fertilization treatments for Mg content was observed only in the root.

### 3.3. Leaf Turnover Rate

**Figure 4:**
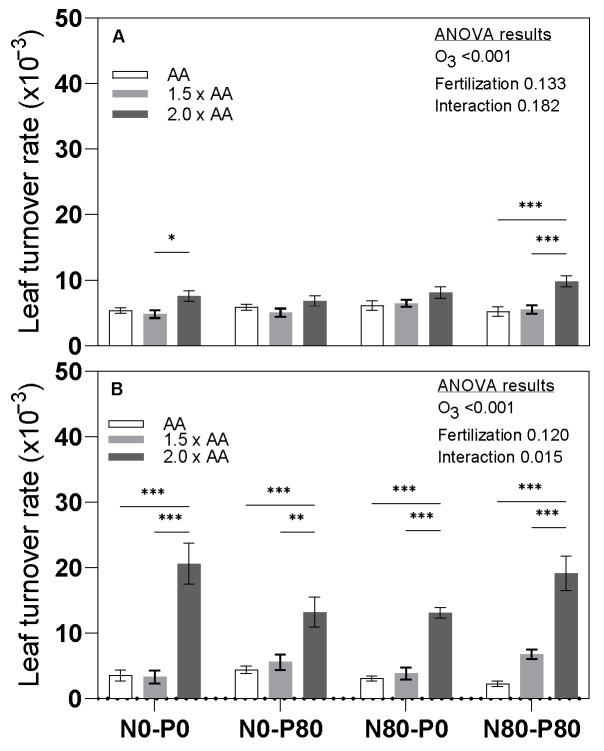
Leaf turnover rate at mid-summer ((**A**), from 18 July to 29 August), and in autumn ((**B**), from 29 August to 23 September) for Oxford poplar clone exposed to three levels of O_3_ (ambient air [AA], 1.5 × AA, 2.0 × AA) with different combinations of soil nutritional conditions (two levels of N [0 and 80 kg N ha^−1^; N0 and N80] and two levels of P [0 and 80 kg P ha^−1^; P0 and P80]). The bars represent mean ± S.E., N0-P0 (AA) *n* = 8, N0-P0 (1.5 × AA) *n* = 7, NO-PO (2.0 × AA) *n* = 9, NO-P80 (AA) *n* = 9, NO-P80 (1.5 × AA) *n* = 9, NO-P80 (2.0 × AA) *n* = 8, N80-PO (AA) *n* = 9, N80-PO (1.5 × AA) *n* = 8, N80-PO (2.0 × AA) *n* = 8, N80-P80 (AA) *n* = 9, N80-P80 (1.5 × AA) *n* = 9, N80-P80 (2.0 × AA) *n* = 9. Asterisks indicate the level of significance of a two-way ANOVA within each group: *** *p* < 0.001, ** *p* < 0.01, * *p* < 0.05. The absence of asterisks indicates that there were no significant differences (*p* > 0.05). The ANOVA table results explain the overall variation, with the level of significance expressed numerically through the *p*-value.

At mid-summer, the leaf turnover rate of N80-P80 (2.0 × AA) exhibited a substantial increase compared to AA, with a rise of +87.4% (Figure 4). Moreover, both N0-P0 (2.0 × AA) and N80-P80 (2.0 × AA) demonstrated higher leaf turnover rates than N0-P0 (1.5 × AA) and N80-P80 (1.5 × AA), with increases of +57.9% and +77.1%, respectively. Interestingly, no interaction was found between O_3_ exposure and fertilization treatments at mid-summer.

In autumn, treatments N0-P0 (2.0 × AA), N0-P80 (2.0 × AA), N80-P0 (2.0 × AA), and N80-P80 (2.0 × AA) displayed significantly higher leaf turnover rates than AA, with increases of +478.9%, +199.2%, +324.8%, and +733.3%, respectively. Furthermore, these treatments also exhibited higher leaf turnover rates compared to their controls with lower phosphorus levels, with increases ranging from +136.7% to +521.9%. Interestingly, a significant interaction between O_3_ exposure and fertilization treatments was observed in autumn.

### 3.4. Principal Component Analysis

**Figure 5:**
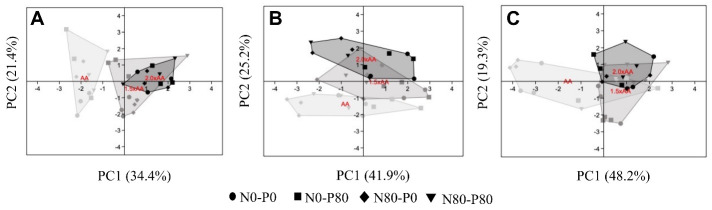
A Principal Component Analysis (PCA) was conducted on all primary and secondary elements present in leaves (**A**), stems (**B**), and roots (**C**).

The PCA displayed the same three distinct groups of populations in any plant organ, and the three groups were the O_3_ treatments, i.e., AA, 1.5 × AA, and 2.0 × AA (Figure 5). However, the distribution of the three organs (leaves, stems, roots) in the PCA was different depending on the organ.

## 4. Discussion

To ensure their existence, plants have to cope with a variety of environmental restrictions such as O_3_ and nutrient availability [37]. Nutrients can generally be divided into two groups, i.e., mobile nutrients which can easily be transported from old leaves to new ones through the phloem, and non-mobile nutrients, such as S and Ca, which can be transported from the roots to leaves through the xylem but have limited mobility in the phloem [38]. Contrary to recent studies by Agathokleous et al. [18], Shang et al. [17], and Wittig et al. [16], which showed that O_3_ exposure usually did not affect the foliar C content, we found a significant increase in this element in the leaves (+3.0% for 1.5 × AA) and an overall significance at the stem level, while the other mobile macronutrients responded similarly to the findings of the above studies. The overall interaction of O_3_ and fertilization for C was found to be significant only for the roots, which is similar to the results observed for the primary macronutrients. N is a constituent of amino acids, amides, proteins, nucleic acids, nucleotides, and coenzymes [39]. Our results confirmed that plants invest additional N to counteract the damage caused by elevated O_3_ levels [40], although the increase was only in the leaves (e.g., +34.2 for 1.5 × AA and +23.7% for 2.0 × AA) and stems (e.g., +54.6% for 1.5 × AA and +48.5% for 2.0 × AA). As pointed out by Shang et al. [41], alterations in N allocation in plant leaves in response to elevated O_3_ can have significant ecological consequences, i.e., leaf litter decomposition. Interestingly, also fertilization resulted in a significant increase in N content in the leaves and stems, but not in the roots. However, the interaction between O_3_ and N was not significant in all anatomical organs. This result, particularly concerning the roots, is consistent with the study published by Ping et al. in 2023 [42]. P is a component of sugar phosphates, nucleic acids, nucleotides, coenzymes, and phospholipids, and plays a key role in reactions that involve ATP [39]. The results revealed significant variations in the average P content among the groups investigated. However, the response exhibited a complex pattern. For instance, exposure to O_3_ led to an increase in P content at the stem level (e.g., +33.7% for 1.5 × AA and +41.1% for 2.0 × AA) and root level (e.g., +25.2% for 1.5 × AA and +59.9% for 2.0 × AA). These findings are in agreement with Shang et al. [17]. Additionally, fertilization with N and P soil enrichments resulted in a significant increase in P content at the stem level (+23.4%). Conversely, at the root level, only the thesis enriched with N displayed an increase in P content under the O_3_ treatment (e.g., +97.2% for 1.5 × AA and +105.7% for 2.0 × AA). The ANOVA revealed a significant overall interaction between O_3_ and P across all anatomical parts investigated. Nonetheless, as emphasized by Shang et al. [17], the growth of poplar plants may be restricted by N rather than P. However, it is worth noting that fertilization can still play a crucial role in modifying ozone-induced effects at the plant’s biochemical level. K is essential as a cofactor for over 40 enzymes, or as a cation to establish cell turgor and maintain cell electroneutrality [39]. The K content was increased in both the leaf and root by the O_3_ treatment (+33.3% and +19.7% for 2.0 × AA, respectively). Overall, it was not influenced by fertilization, except for a significant interaction of the two factors observed only in the roots. The increase in potassium (K) observed in the plants treated with O_3_ is consistent with findings from other studies, such as Leone [43]. This rise in K levels in plants is certainly attributed to a reduction in stomatal resistance, where it is known that this element plays a role in regulating the stomatal opening. Overall, our findings suggest that the O_3_ treatment increased the contents of C (in the leaf), N (in the stem), P (in the stem and root), K (in the leaf and root), Ca (in the root), S (in the leaf), and Mg (in the leaf and root), while decreasing the contents of C and S only at the root level. Similar to our findings, O_3_ exposure is often linked to increased mineral concentration in plants [44], which is commonly attributed to a growth concentration effect (i.e., higher concentration of an element at lower biomass yield). Shang et al. [38] highlighted that elevated O_3_ enhanced the accumulation of Ca in older leaves of two poplar clones relative to the younger leaves. Ca is required as a cofactor by some enzymes involved in the hydrolysis of ATP and phospholipids and acts as a second messenger in metabolic regulation [39]. Nevertheless, we found a significant decrease in Ca in leaves following O_3_ exposure (−19% for 1.5 × AA relative to AA). Our different outcome could be explained as the result of leaf sampling, as we mixed up all leaves and then collected three randomized sub-samples thus including both younger and older leaves. Non-mobile nutrients such as Ca usually show lower concentrations in upper leaves than in lower leaves [38]. Thus, another reason for the discrepancy between our result for Ca and that of Shang et al. [38] may be our accelerated leaf turnover rate due to O_3_ exposure, which reduced the proportion of lower leaves in our mixed samples. In addition, we cannot exclude clone-specific stoichiometric responses to O_3_ exposure, as found for the photosynthetic responses in poplar [45] and beech [46]. The decrease in Ca content in the leaves was confirmed by a decrease in the stem (−18.9% N0-P80 2.0 × AA relative to AA), while the content increased in the roots, which is consistent with the reduced mobility of this nutrient [38] and the O_3_-induced reduction of stomatal conductance in this clone as reported in our previous paper [13]. A positive significant result about the fertilization was mainly found at the root level with the thesis fertilized by N or P with respect to AA. Fertilization could have potentially enhanced root growth and nutrient uptake. In agreement with recent findings [17,23], no interactive effects of O_3_ and fertilization on foliar Ca content were found, while the interaction was significant in the root, suggesting that both fertilization and ozone can enhance the allocation of this element in this anatomical part. Sulfur is a component of cysteine, cystine, methionine, and proteins, as well as lipoic acid, coenzyme A, thiamine pyrophosphate, glutathione, biotin, adenosine-5’-phosphosulfate, and 3-phosphoadenosine [39]. The exposure to O_3_ had a significant impact on S, with a noticeable increase of 33.6% in the leaves for 2.0 × AA compared to AA, and a decrease of 20.2% in the roots for 1.5 × AA compared to AA. The findings pertaining to leaves are surprising, as the results contradict the expectation for this non-mobile nutrient [47]. However, there was no variation in S due to leaf-level fertilization with respect to AA. Interestingly, the effect was significant at the stem and root levels. At the root level, a well-balanced fertilization treatment facilitated increased absorption of sulfur (S) when plants were exposed to ozone. The interaction between O_3_ and fertilization was mainly significant at the root level, confirming the capacity of fertilization to reduce the negative O_3_ impacts on plants [37]. Mg content, which is commonly required by many enzymes involved in phosphate transfer or is part of the constituent of the chlorophyll molecule [39], significantly increased following O_3_ exposure in leaves (+26.1% for 2.0 × AA) and roots (+46.9% for 1.5 × AA and +56.8% for 2.0 × AA), in agreement with other species [48]. Also, fertilization significantly changed the overall variation of the Mg contents of all plant organs. The overall interaction of O_3_ and fertilization for Mg was significant only for the roots, as the contents under 2.0 × AA O_3_ exposure were higher than in the AA control group only for the unfertilized plants, still supporting an ameliorative capacity of fertilization against the O_3_ impacts on stoichiometric imbalances. Ozone exposure can trigger leaf abscission and turnover in tree species with continuous leaf emergence such as poplar [49], birch [50], and oak [51]. Likewise, our study found that O_3_ exposure significantly accelerated the rate of leaf turnover, which may be a plant strategy to counteract oxidative damage by replacing damaged leaves with new productive leaves. A highly significant impact of the O_3_ treatment on leaf turnover rate is particularly clear in Figure 4 (graph B). However, it should be noted that O_3_ impairs the nutrient resorption capacity of old leaves as reported in birch [52] and poplars [17]. Overall, our results also revealed a noteworthy interaction between fertilization and O_3_ in impacting the leaf turnover rate in plants. This also suggests that the effect of O_3_ and fertilization on leaf turnover rate was different in the two phases investigated. This phenomenon can be attributed to the new leaves’ capacity to respond to the stress. Young leaves typically have more responsive stomata compared to older leaves. Furthermore, the uptake of elements from the fertilized soil may also contribute to this response, providing the plants with an increased supply of minerals for support. The PCA analysis displayed three distinct population groups across the plant organs under investigation when considering all nutrients together. Specifically, the PCA analysis showed that the control group (AA), which also received fertilization, is notably distinct from the other two populations (1.5 × AA and 2.0 × AA). This suggests that O_3_ fumigation primarily affects the overall allocation of primary and secondary elements, as demonstrated in the literature (e.g., [17]). This assertion can be supported by considering that fertilization is also applied in some AA groups. When studying all nutrients together, the effect of O_3_ treatment on plants becomes particularly evident in the leaves, where the control group is completely distinct from the other one. This result can be interpreted as an attempt by the plant to redistribute the available elements, including those from fertilization, at the leaf level. However, a similar distribution pattern among the three groups is also noticeable in the stem and root, albeit with more clarity. This difference could be attributed to the varying levels of exposure of this organ to the fumigation.

## 5. Conclusions

For the first time, the results provide information on the primary and secondary macronutrients under different fertilization treatments and exposure to O_3_ in poplar during a New-Generation 3D Ozone FACE (Free Air Controlled Exposure) experiment. Overall, our findings suggest that the O_3_ treatment increased the contents of C (in the leaf), N (in the stem), P (in the stem and root), K (in the leaf and root), Ca (in the root), S (in the leaf), and Mg (in the leaf and root), while decreasing the contents of C and S only at the root level. In general, at the single element level, the results showed that fertilization can have a significant effect on the nutrient content in the plant, with some treatments leading to higher nutrient content and others leading to lower content compared to the control group. Overall, these results suggest that both fertilization and O_3_ exposure can significantly affect leaf turnover rate in plants, and the effect can vary depending on the level of exposure and the phase of the experiment.

## Data Availability

Data are contained within the article.
